# The National Hospital

**Published:** 1905-03-25

**Authors:** 


### The Hospital.

A Journal of

The Medical Sciences and Hospital Administration.

Vol. XXXVII.—No. 965. MARCH 25, 1905.

### THE NATIONAL HOSPITAL.

If any justification were required of the daily increasing demand for detailed economy in the management of hospitals, it would be furnished by a very remarkable correspondence which has passed between the chairman of committee of the National Hospital for the Paralysed and Epileptic, Mr. Danvers Power, and Sir Edmund Hay Currie as secretary of the Hospital Sunday Fund. The attention of Mr. Power's Committee was directed to questions of domestic economy by a circular which was received in the middle of 1903 from the Committee of the Hospital Sunday Fund, addressed to them and to thirty-seven other hospitals, and announcing that the expenditure on in-patients was too large, and that, until it was reduced, the grant from the Fund would be made upon a lower basis. As far as we are informed, the other thirty-seven Committees have made no sign; but the Committee of the National, although the alleged excess was much less in their case than in some others, at once set themselves to work to find a remedy for the fault to which their attention had been directed, and the letter from their Chairman now announces that they have been successful. They evidently had among their number counsellors as wise as those who deprecated the wrath of Naaman the Syrian. They did not set themselves to accomplish any "great thing," but simply to look for possible economies, individually small, but in the aggregate considerable.

They appointed a Committee, consisting of their own matron and half-a-dozen other ladies connected with hospitals and Poor-law institutions, and requested them to inquire closely into every item of expenditure, as well as into the incidents of consumption connected with the provisioning and domestic arrangements of the hospital. By their advice the period of meat contracts was lengthened, and town-killed foreign beef was substituted for English beef; the prices of fish, fowls, bacon, and milk were reduced, and a method of purchasing vegetables and fruit, based on taking daily advantage of the condition of the market, was introduced. The ladies made further important recommendations respecting the handling and distribution of goods received into the hospital. They showed that the system of cutting bread for the patients occasioned waste; that the cooking ovens were not of the kind best suited for the work required of them; that the employment of a skilled carver in the kitchen, instead of carving joints in the wards, would effect large economies; that much cheaper crockery than was being used would answer all ordinary purposes; that some of the electric light lamps were of unnecessarily high candle-power and badly distributed; and, finally, that it would be more economical in every way to employ a female housekeeper instead of a steward. Mr. Danvers Power states that when the new board assumed office in 1902 the cost per bed went up from £84 to £97. The adoption of these recommendations, however, reduced the expenses per bed to £81 0s. 1d. in 1904, or by almost one-sixth; and, while the Medical Committee has declared that the staff have been "perfectly satisfied with the nursing and other arrangements," the report made to the Hospital Sunday Fund has drawn from Sir Edmund Hay Currie the declaration that, if the other 37 hospitals which received a similar intimation had acted upon it in an equally effectual manner, the resulting saving would have amounted to £30,000 a year, or to the interest of one million sterling.

The moral of the story is, of course, as old as the proverb which declares that, if we take care of the pence the pounds will take care of themselves. The sources of unnecessary expenditure in hospitals are here a little and there a little; and it is probable that committees, revolving in their minds great schemes of rebuilding or of extension, may sometimes be too apt to leave the close supervision of expenditure in the hands of subordinates who have no particular interest in the matter, or who, in some cases, may possibly not be beyond the reach of the temptations which too many tradespeople are apt to put in the way of the domestics of a family. It is evident that the only way of dealing with the evils hence arising is, in the first place by the adoption of the uniform method of keeping accounts on which Sir Henry Burdett has so long insisted, and then by a rigid determination that no excessive expenditure in any particular shall be overlooked. Many years ago, when an eminent surgeon, now deceased, was advocating something not very far removed from constant intoxication as a sovereign remedy for most diseases, he was accustomed to declare, in clinical lectures and in communications to the medical journals, that the efficacy of his treatment might have remained unknown were it not that he had the good fortune to be on the staff of a rich hospital, where the amount of his wine bill was a matter of indifference to the management. Even he would to-day be unable to dwell upon this topic of congratulation ; and it is at least conceivable that the actual financial condition of most hospitals may not be an unmixed evil, if it compels those who are mainly responsible for expenditure to think twice before they authorise anything that is unusual.

Of the total saving announced by Mr. Danvers Power, about £200 was effected in the surgery and dispensary; and it may be questioned whether some physicians and surgeons are not too ready to surrender their judgments to the numerous manufacturing chemists whose business it is to place new and costly (play) things, mostly "made in Germany," upon the market. Sir Astley Cooper, a very wise man in his generation, was accustomed to warn students against new remedies, and to advise them to learn what could be done with the old ones. "If," he said, "you are too fond of new remedies, two consequences will follow. The first will be that you will not cure your patients, the second, that you will soon have no patients to cure."

